# Funcionalidade da HDL, Inibidores de CETP e Treinamento Físico: Onde Estamos?

**DOI:** 10.36660/abc.20260145

**Published:** 2026-06-10

**Authors:** Raul D. Santos, Pedro Gabriel Senger Braga

**Affiliations:** 1 Hospital Israelita Albert Einstein São Paulo SP Brasil Hospital Israelita Albert Einstein, São Paulo, SP – Brasil; 2 Universidade de São Paulo São Paulo SP Brasil Universidade de São Paulo, São Paulo, SP – Brasil; 3 Clínica Pró-Coração São Paulo SP Brasil Clínica Pró-Coração, São Paulo, SP – Brasil; 4 Instituto do Coração do Hospital das Clínicas da Faculdade de Medicina da Universidade de São Paulo Departamento de Cardio-Oncologia São Paulo SP Brasil Departamento de Cardio-Oncologia – Instituto do Coração do Hospital das Clínicas da Faculdade de Medicina da Universidade de São Paulo, São Paulo, SP – Brasil

**Keywords:** HDL-Colesterol, Proteínas de Transferência de Ésteres de Colesterol, Exercício Físico

O treinamento físico é bem consolidado como uma estratégia eficaz para aumentar as concentrações de colesterol da lipoproteína de alta densidade (HDL-C), especialmente em pessoas com triglicerídeos elevados e excesso de adiposidade visceral, sendo consistentemente associado a menor risco de doenças cardiovasculares (DCV).^1^ No entanto, a estratificação tradicional do risco de DCV baseada exclusivamente nos níveis de HDL-C tem sido cada vez mais questionada por evidências que destacam a importância de avaliar a funcionalidade da HDL, e não apenas os níveis de HDL-C. Essa mudança de paradigma ganhou particular atenção após os ensaios clínicos com inibidores da proteína de transferência de ésteres de colesterol (CETP), nos quais aumentos substanciais de HDL-C mediados farmacologicamente não se traduziram em melhores desfechos cardiovasculares. Os ensaios com inibidores de CETP relataram elevações acentuadas de HDL-C, variando de aproximadamente 31% a 133%, enquanto o treinamento físico normalmente induz um aumento mais modesto, da ordem de 2% a 5%.^2–5^ Apesar dessas diferenças quantitativas, ambas as intervenções demonstraram melhorar aspectos da funcionalidade das partículas de HDL. Em intervenções baseadas em treinamento físico, os aumentos de HDL-C são acompanhados por melhorias na captação de colesterol mediada pela HDL, comumente avaliada pela capacidade de efluxo de colesterol (CEC), uma métrica que reflete com mais precisão o transporte reverso de colesterol e o risco de DCV, em comparação com a concentração de HDL-C isoladamente.^6–10^ Mecanisticamente, a inibição da CETP também aumenta a CEC da HDL, indicando que as partículas de HDL farmacologicamente aumentadas podem reter propriedades funcionais. No entanto, efeitos adversos fora do alvo e a modulação artificial do metabolismo das lipoproteínas podem explicar parcialmente os desfechos cardiovasculares neutros ou desfavoráveis observados em diversos ensaios com inibidores de CETP.^2,4,5^ Em conjunto, esses achados ressaltam que, embora tanto o treinamento físico quanto a inibição de CETP aumentem substancialmente o HDL-C em circulação, o contexto biológico, os efeitos qualitativos nas partículas de HDL e as implicações clínicas diferem fundamentalmente entre essas intervenções, reforçando a necessidade de ir além da quantidade de HDL-C ao avaliar o risco cardiovascular e as estratégias terapêuticas.

## Lipoproteína de alta densidade e treinamento físico

O treinamento físico está associado a um aumento modesto, porém consistente, do HDL-C, tipicamente de aproximadamente 2 a 5 mg/dL, conforme demonstrado em ensaios randomizados e meta-análises.^11,12^ Embora quantitativamente limitado em comparação com abordagens farmacológicas, esse aumento é fortemente influenciado pela dose, intensidade e duração do treinamento, com volumes maiores e exercícios aeróbicos de alta intensidade produzindo efeitos mais pronunciados, incluindo redução do peso corporal e dos níveis de triglicerídeos.^7^ Mecanisticamente, o exercício aumenta a atividade da lipase lipoproteica no músculo esquelético, aumenta a produção de apolipoproteína A-I e melhora o fluxo de transporte reverso de colesterol, em vez de simplesmente aumentar o conteúdo de colesterol dentro das partículas de HDL. Vários estudos clínicos demonstraram que os aumentos do HDL-C induzidos pelo exercício são frequentemente acompanhados por melhorias na funcionalidade da HDL, especialmente na CEC. Nos ensaios STRRIDE-PD e E-MECHANIC, apenas o treinamento de alta dose ou de intensidade vigorosa melhorou significativamente a CEC radiomarcada, sugerindo um efeito limiar para o benefício funcional.^7^ Outras intervenções relataram melhorias no efluxo mediado pelo transportador de cassete de ligação ao ATP A1 (ABCA1), propriedades anti-inflamatórias endoteliais e capacidade antioxidante da HDL, embora os resultados tenham variado de acordo com as características da população e os ensaios experimentais.^8–10,13^

Um estudo de coorte prospectivo dentro do Henry Ford Exercise Testing Project (FIT Project) investigou a relação entre HDL-C baixo isolado, aptidão cardiorrespiratória e mortalidade a longo prazo em adultos. Nesta análise, indivíduos com apenas HDL-C baixo (definido como HDL-C < 40 mg/dL para homens e < 50 mg/dL para mulheres) e LDL-C e triglicerídeos < 100 mg/dL apresentaram aptidão média significativamente menor e mortalidade maior durante um acompanhamento médio de 10,3 ± 5 anos em comparação com aqueles com perfis lipídicos otimizados.^1^ A mortalidade foi particularmente elevada entre os participantes com HDL-C baixo isolado que atingiram menos de 6 equivalentes metabólicos (METs) durante o teste de exercício, com razões de risco ajustadas de 1,73 (intervalo de confiança de 95% 1,18–2,54) e 1,90 (intervalo de confiança de 95% 1,19–3,04) para as categorias < 6 e 6–10 METs, respectivamente. Não foi observada diferença significativa entre aqueles que atingiram ≥ 10 METs.^14^ Esses resultados ressaltam que, embora o HDL-C baixo isolado esteja associado a um risco aumentado de mortalidade, uma melhor aptidão cardiorrespiratória atenua esse risco, destacando a interação crítica entre aptidão física e fenótipos lipídicos na estratificação do risco cardiovascular.^12^

Esses achados sugerem que o exercício melhora tanto a quantidade quanto a qualidade da HDL, mesmo quando as mudanças absolutas no HDL-C são modestas, conforme mostrado na Tabela 1. É importante ressaltar que essas adaptações específicas da HDL ocorrem juntamente com melhorias cardiometabólicas mais amplas, incluindo maior sensibilidade à insulina, redução dos triglicerídeos, diminuição da pressão arterial e redução da inflamação sistêmica.

**Tabela 1:** Estudos clínicos de treinamento físico que avaliaram a funcionalidade da HDL em seres humanos

Estudo (ano)	População e delineamento	Principais achados funcionais	Principais implicações
Sarzynski et al., ATVB (2018)^7^	Ensaios randomizados de exercício; adultos com pré-diabetes/sobrepeso (STRRIDE-PD) e adultos com sobrepeso/obesidade (E-MECHANIC); 6 meses; dose e intensidade variáveis	STRRIDE-PD: O efluxo radiomarcado global aumentou ~6,2% apenas no grupo de alta quantidade/intensidade vigorosa. E-MECHANIC: O efluxo não mediado por ABCA1 aumentou ~5,7% no grupo de dose mais alta (20 kcal/kg/semana) versus controle; nenhuma alteração consistente no efluxo mediado por ABCA1.	Sugere um limiar de dose/intensidade: treinamento de resistência prolongado e vigoroso/ de alta dose melhoram as medidas de efluxo.
Stanton et al., JAHA (2022)^8^	Homens jovens e saudáveis; treinamento sequencial supervisionado de intensidade moderada e, em seguida, de alta intensidade (desenho intraindividual).	O efluxo mediado por ABCA1 aumentou 13,5% após o treinamento de intensidade moderada e permaneceu elevado em 17,3% após o treinamento subsequente de alta intensidade.	O exercício pode melhorar o efluxo específico de ABCA1, com benefícios mantidos após maior intensidade.
Boyer et al., Am J Physiol Endocrinol Metab (2018)^13^	Homens com obesidade abdominal e dislipidemia; programa de estilo de vida de 1 ano (dieta saudável + aumento da atividade física/exercício) versus controle	J774-HDL-CEC aumentou 14,1%; HepG2-HDL-CEC aumentou 3,4%; sem alterações no grupo controle; as melhorias foram acompanhadas por alterações na apolipoproteína A-I.	Mudanças sustentadas no estilo de vida (incluindo exercícios) melhoram a capacidade de efluxo de HDL na obesidade abdominal dislipidêmica.
Woudberg et al., Lipids in Health and Disease (2018)^9^	Mulheres obesas; intervenção com exercícios versus controle	O exercício não alterou a capacidade de efluxo celular da HDL.	O exercício não afetou o efluxo da HDL.
Zhu et al., Frontiers in Physiology (2024)^10^	Adultos magros, obesos não diabéticos e obesos com DM2; programa HIIT de curta duração (15 dias)	A capacidade de efluxo melhorou apenas nos indivíduos magros.	As alterações na função da HDL relacionadas ao HIIT parecem depender do contexto metabólico.

ABCA1: transportador de cassete de ligação ao ATP A1; CEC: capacidade de efluxo de colesterol; DM2: diabetes mellitus tipo 2; HIIT: treinamento intervalado de alta intensidade; J774: linhagem celular de macrófagos; J774-HDL-CEC: ensaio laboratorial que mede a capacidade de efluxo de colesterol.

## Lipoproteína de alta densidade e inibição da proteína de transferência de ésteres de colesterol

Em contraste, os inibidores de CETP produzem elevações acentuadas no HDL-C, muito maiores do que as alcançadas com intervenções no estilo de vida.^2,5,15,16^ Torcetrapib, dalcetrapib, evacetrapib e anacetrapib aumentam o HDL-C principalmente pela inibição da transferência de ésteres de colesterol da HDL para lipoproteínas contendo apolipoproteína B, resultando em partículas de HDL maiores e enriquecidas em colesterol. Estudos mecanísticos em seres humanos demonstraram que a inibição de CETP geralmente melhora as métricas funcionais da HDL, incluindo a CEC total e a CEC mediada por ABCA1, e aumenta as concentrações de HDL pré-β1, um importante receptor inicial de colesterol celular.^17,18^ Esses achados mitigaram as preocupações iniciais de que a HDL induzida pela inibição de CETP pudesse ser disfuncional. No entanto, apesar da CEC preservada ou aumentada, a maioria dos inibidores da CETP não conseguiu reduzir eventos cardiovasculares em grandes ensaios clínicos. O torcetrapib aumentou os eventos cardiovasculares e a mortalidade por todas as causas devido a efeitos colaterais, incluindo a ativação da aldosterona e a elevação da pressão arterial.^2^ O dalcetrapib e o evacetrapib foram clinicamente neutros, apesar dos aumentos substanciais no HDL-C e das capacidades funcionais favoráveis.^5,16^ Conforme resumido na Tabela 2, apenas o anacetrapib demonstrou uma redução modesta nos principais eventos coronários, um efeito atribuído principalmente à redução não HDL, e não à magnitude da elevação do HDL-C em si. Atualmente, o obicetrapib, um novo inibidor de CETP, está sendo testado em pacientes com LDL-C que permanece elevado, apesar da terapia hipolipemiante, com o objetivo de prevenir eventos cardiovasculares não pelo aumento do HDL-C, mas pela redução das lipoproteínas pró-aterogênicas que contêm apolipoproteína B (Estudo PREVAIL, ClinicalTrials.gov NCT05202509).

**Tabela 2:** Inibidores de CETP e funcionalidade da HDL em humanos

Medicamento (estudo)	População	Principais achados funcionais	Principais implicações
Torcetrapib – estudos mecanísticos em seres humanos (Yvan-Charvet et al.)^18^	Indivíduos com hipercolesterolemia	Aumento moderado da CEC com 60 mg; aumento maior com 120 mg	A funcionalidade da HDL melhorou, mas isso não se traduziu em benefício clínico devido a efeitos adversos.
Dalcetrapib – dal-ACUTE	Pacientes pós-SCA	Aumento do HDL-C e da apolipoproteína A-I com melhora no efluxo de colesterol	Foi observada melhora funcional da HDL em contexto inflamatório.
Dalcetrapib – análise de genótipo (Tardif et al.)^17^	Pacientes pós-SCA estratificados por genótipo	Melhora da capacidade de efluxo apenas em genótipos específicos	A resposta da funcionalidade da HDL é geneticamente determinada.
Evacetrapib – subestudo mecanístico	Pacientes com dislipidemia	Aumento significativo do efluxo total e específico para ABCA1	Houve melhora significativa da funcionalidade da HDL apesar de desfechos clínicos neutros.
Anacetrapib – DEFINE subestudo mecanístico	Pacientes com doença arterial coronariana	Aumento consistente da CEC.	A melhora funcional da HDL foi associada a um benefício clínico modesto.

CEC: capacidade de efluxo de colesterol; SCA: síndrome coronariana aguda.

## Quantidade versus qualidade

Em conjunto, esses dados reforçam o conceito de que alterações na concentração de HDL-C, por si só são um biomarcador substituto inadequado para proteção cardiovascular. O exercício induz aumentos menores no HDL-C, mas promove uma melhora integrada da funcionalidade da HDL e na saúde cardiometabólica sistêmica, enquanto a inibição de CETP produz grandes aumentos no HDL-C com benefício clínico inconsistente. Notavelmente, as melhorias na função da HDL observadas com o exercício ocorrem em um ambiente fisiológico caracterizado por inflamação reduzida e melhora da função endotelial, enquanto a inibição de CETP tem como alvo uma única via metabólica. Essa distinção pode explicar por que os aumentos no HDL-C associados ao exercício estão consistentemente ligados a benefícios cardiovasculares, enquanto as elevações no HDL-C induzidas farmacologicamente não estão.

## Implicações clínicas

A partir de uma perspectiva clínica e translacional, esses achados sugerem que aumentar o HDL-C por si só não deve ser um objetivo terapêutico primário, a menos que seja acompanhado por melhorias demonstráveis na funcionalidade da HDL e no risco cardiovascular geral. O exercício continua sendo uma intervenção singularmente eficaz, pois melhora a função da HDL de maneira biologicamente integrada, mesmo quando as alterações no HDL-C são modestas. Por outro lado, a experiência com os inibidores de CETP destaca as limitações de se visar os níveis de HDL-C sem compreender completamente seus papéis funcionais e sistêmicos. Em resumo, o exercício produz efeitos menores, porém mais coerentes fisiologicamente e clinicamente significativos, enquanto os inibidores de CETP evidenciam uma dissociação entre a elevação do HDL-C, a funcionalidade da HDL e os desfechos cardiovasculares. Embora o veredito final sobre os inibidores de CETP ainda esteja pendente, o exercício deve ser prescrito como um pilar da prevenção de DCV, devido aos seus efeitos benéficos sobre a pressão arterial e o metabolismo da glicose, reduzindo assim o risco cardiovascular.
