# Supplementary material for: Funcionalidade da HDL, Inibidores de CETP e Treinamento Físico: Onde Estamos?
Source: Arq Bras Cardiol. 2026 Jun 10;123(5):e20260145. [Article in Portuguese] doi: 10.36660/abc.20260145 (PMC13399269; doi:10.36660/abc.20260145)
Supplement: Translation

# Translation: HDL Functionality, CETP Inhibitors, and Exercise Training: Where are We?

**DOI:** 10.36660/abc.20260145i

**Authors:** Raul D. Santos, Pedro Gabriel Senger Braga

**Affiliations:** 1 São Paulo SP Brazil Hospital Israelita Albert Einstein, São Paulo, SP – Brazil; 2 São Paulo SP Brazil Universidade de São Paulo, São Paulo, SP – Brazil; 3 São Paulo SP Brazil Clínica Pró-Coração, São Paulo, SP – Brazil; 4 São Paulo SP Brazil Departamento de Cardio-Oncologia – Instituto do Coração do Hospital das Clínicas da Faculdade de Medicina da Universidade de São Paulo, São Paulo, SP – Brazil

**Keywords:** HDL Cholesterol, Cholesterol Ester Transfer Proteins, Exercise

Exercise training is well established as an effective strategy to increase high-density lipoprotein cholesterol (HDL-C) concentrations, especially in people with high triglycerides and excess visceral adiposity, and is consistently associated with a lower risk of cardiovascular disease (CVD).^1^ However, the traditional stratification of CVD risk based solely on HDL-C levels has been increasingly challenged by evidence highlighting the importance of assessing HDL particle functionality rather than HDL-C levels alone. This paradigm shift gained particular attention following clinical trials of cholesteryl ester transfer protein (CETP) inhibitors, in which substantial pharmacologically mediated increases in HDL-C failed to translate into improved cardiovascular outcomes. CETP inhibitor trials reported marked elevations in HDL-C, ranging from approximately 31% to 133%, whereas exercise training typically induces a much more modest increase, on the order of 2% to 5%.^2–5^ Despite these quantitative differences, both interventions have been shown to enhance aspects of HDL functionality. In exercise-based interventions, increases in HDL-C are accompanied by improvements in HDL-mediated cholesterol uptake, commonly assessed by cholesterol efflux capacity (CEC), a metric that more accurately reflects reverse cholesterol transport and CVD risk than HDL-C concentration alone.^6–10^ Mechanistically, CETP inhibition also enhances HDL cholesterol efflux capacity, indicating that pharmacologically enlarged HDL particles may retain functional properties. Nevertheless, adverse off-target effects and the artificial modulation of lipoprotein metabolism may partly explain the neutral or unfavorable cardiovascular outcomes observed in several CETP inhibitor trials.^2,4,5^ Together, these findings underscore that, although both exercise training and CETP inhibition substantially increase circulating HDL-C, the biological context, qualitative effects on HDL particles, and clinical implications differ fundamentally between these interventions, reinforcing the need to move beyond HDL-C quantity when evaluating cardiovascular risk and therapeutic strategies.

## High-density lipoprotein and exercise training

Exercise training is associated with a modest but consistent increase in HDL-C, typically 2 to 5 mg/dL, as demonstrated in randomized trials and meta-analyses.^11,12^ Although quantitatively limited compared with pharmacological approaches, this increase is strongly influenced by exercise dose, intensity, and duration, with higher volumes and vigorous-intensity aerobic exercise yielding more pronounced effects, including reduced body weight and triglyceride levels.^7^ Mechanistically, exercise enhances skeletal muscle lipoprotein lipase activity, increases apolipoprotein A-I production, and improves reverse cholesterol transport flux rather than merely increasing cholesterol content within HDL particles. Several clinical studies have demonstrated that exercise-induced increases in HDL-C are often accompanied by improvements in HDL functionality, particularly CEC. In the STRRIDE-PD and E-MECHANIC trials, only high-dose or vigorous-intensity exercise significantly improved radiolabeled CEC, suggesting a threshold effect for functional benefit.^7^ Other interventions have reported improvements in ATP-binding cassette transporter A1 (ABCA1)-mediated efflux, endothelial anti-inflammatory properties, and HDL antioxidant capacity, although results varied by population characteristics and experimental assays.^8–10,13^

A prospective cohort study within the Henry Ford Exercise Testing Project (FIT Project) investigated the relationship between isolated low HDL-C, cardiorespiratory fitness, and long-term mortality in adults. In this analysis, individuals with isolated low HDL-C (defined as HDL-C < 40 mg/dL for men and < 50 mg/dL for women) and LDL-C and triglycerides < 100 mg/dL exhibited significantly lower mean fitness and higher mortality over a mean follow-up of 10.3 ± 5 years compared with those with optimal lipid profiles.^1^ Mortality was particularly elevated among participants with isolated low HDL-C who achieved less than 6 metabolic equivalents (METs) during exercise testing, with adjusted hazard ratios of 1.73 (95% confidence interval 1.18–2.54) and 1.90 (95% confidence interval 1.19–3.04) for < 6 and 6–10 MET categories, respectively; no significant difference was observed among those attaining ≥ 10 METs.^14^ These results underscore that, while isolated low HDL-C is associated with increased mortality risk, higher cardiorespiratory fitness attenuates this risk, highlighting the critical interplay between fitness and lipid phenotypes in cardiovascular risk stratification.^12^

These findings suggest that exercise improves both the quantity and quality of HDL, even when absolute changes in HDL-C are modest, as shown in Table 1. Importantly, these HDL-specific adaptations occur alongside broader cardiometabolic improvements, including enhanced insulin sensitivity, reduced triglycerides, lower blood pressure, and decreased systemic inflammation.

**Table 1:** Clinical exercise studies assessing HDL functionality in humans

Study (year)	Population and design	Main functional results	Key interpretation
Sarzynski et al., ATVB (2018)^7^	Randomized exercise trials; prediabetes/overweight adults (STRRIDE-PD) and overweight/obese adults (E-MECHANIC); 6 months; varying dose/intensity	STRRIDE-PD: global radiolabeled efflux ↑ ~6.2% only in the high-amount/vigorous-intensity group. E-MECHANIC: non-ABCA1 efflux ↑ ~5.7% in the highest dose group (20 kcal/kg/week) versus control; no consistent change in ABCA1 efflux.	Suggests a dose/intensity threshold: prolonged vigorous/high-dose endurance training improves efflux measures.
Stanton et al., JAHA (2022)^8^	Healthy young men; supervised sequential moderate- then high-intensity training (within-subject design)	ABCA1-mediated efflux ↑ 13.5% after moderate-intensity training and remained ↑ 17.3% after subsequent high-intensity training.	Exercise can improve ABCA1-specific efflux, with benefits maintained after higher intensity
Boyer et al., Am J Physiol Endocrinol Metab (2018)^13^	Men with abdominal obesity and dyslipidemia; 1-year lifestyle program (healthy diet + increased physical activity/exercise) versus control	J774-HDL-CEC ↑ 14.1%; HepG2-HDL-CEC ↑ 3.4%; no change in controls; improvements tracked with apolipoprotein A-I changes.	Sustained lifestyle change (including exercise) improves HDL efflux capacity in dyslipidemic abdominal obesity.
Woudberg et al., Lipids in Health and Disease (2018)^9^	Obese women; exercise intervention versus control	Exercise did not change HDL cellular efflux capacity.	Exercise did not affect HDL efflux.
Zhu et al., Frontiers in Physiology (2024)^10^	Lean, obese nondiabetic, and obese T2DM adults; short-term HIIT program (15 days)	Efflux capacity improved only in lean subjects.	HIIT-related changes in HDL function appear metabolically context-dependent.

ABCA1: ATP-binding cassette transporter A1; CEC: cholesterol efflux capacity; T2DM: type 2 diabetes mellitus; HIIT: high-intensity interval training; J774: macrophages cell line; J774-HDL-CEC: laboratory assay that measures cholesterol efflux capacity.

## High-density lipoprotein and cholesteryl ester transfer protein inhibition

In contrast, CETP inhibitors produce marked elevations in HDL-C far greater than those achieved with lifestyle interventions.^2,5,15,16^ Torcetrapib, dalcetrapib, evacetrapib, and anacetrapib increase HDL-C primarily by inhibiting the transfer of cholesteryl esters from HDL to apolipoprotein B-containing lipoproteins, resulting in cholesterol-enriched, larger HDL particles. Mechanistic studies in humans have demonstrated that CETP inhibition generally improves HDL functional metrics, including total and ABCA1-mediated CEC, and increases concentrations of pre-β1 HDL, a key initial acceptor of cellular cholesterol.^17,18^ These findings countered early concerns that CETP inhibitor-induced HDL might be dysfunctional. However, despite preserved or enhanced efflux capacity, most CETP inhibitors failed to reduce cardiovascular events in large outcome trials. Torcetrapib increased cardiovascular events and all-cause mortality due to off-target effects, including aldosterone activation and blood pressure elevation.^2^ Dalcetrapib and evacetrapib were clinically neutral despite substantial increases in HDL-C and favorable functional signals.^5,16^ As shown in Table 2, only anacetrapib demonstrated a modest reduction in major coronary events, an effect largely attributed to reductions in non-HDL cholesterol rather than the magnitude of HDL-C elevation itself. Currently, obicetrapib, a novel CETP inhibitor, is being tested in patients with still-elevated LDL-C despite lipid-lowering therapy, with the aim of preventing CVD events not by raising HDL-C but by reducing apolipoprotein B-containing pro-atherogenic lipoproteins (PREVAIL Study, ClinicalTrials.gov NCT05202509).

**Table 2:** ICETP inhibitors and HDL functionality in humans

Drug (study)	Population	Main functional results	Key interpretation
Torcetrapib – mechanistic human studies (Yvan-Charvet et al.)^18^	Hypercholesterolemic subjects	Modest increase in CEC at 60 mg; greater increase at 120 mg	HDL functionality improved, but did not translate into clinical benefit due to off-target effects
Dalcetrapib – dal-ACUTE	Patients after ACS	Increase in HDL-C and apolipoprotein A-I with improved cholesterol efflux	HDL functional improvement observed in inflammatory setting.
Dalcetrapib – genotype analysis (Tardif et al.)^17^	Post-ACS patients stratified by genotype	Improved efflux capacity in specific genotypes only	HDL functionality response is genetically determined.
Evacetrapib – mechanistic substudy	Patients with dyslipidemia	Significant increase in total and ABCA1-specific efflux	Marked improvement in HDL functionality despite neutral clinical outcomes.
Anacetrapib – DEFINE mechanistic substudy	Patients with coronary artery disease	Consistent increase in CEC	Functional HDL improvement aligned with modest clinical benefit.

ACS: acute coronary syndrome; CEC: cholesterol efflux capacity.

## Quantity versus quality

Collectively, these data reinforce the concept that changes in HDL-C concentration alone are an inadequate surrogate biomarker for cardiovascular protection. Exercise induces smaller increases in HDL-C but promotes a coordinated improvement in HDL functionality and systemic cardiometabolic health, whereas CETP inhibition produces large increases in HDL-C with inconsistent clinical benefit. Notably, the improvements in HDL function observed with exercise occur within a physiological milieu characterized by reduced inflammation and improved endothelial function, whereas CETP inhibition targets a single metabolic pathway. This distinction may explain why exercise-associated increases in HDL-C are consistently linked to cardiovascular benefits, whereas pharmacologically induced elevations in HDL-C are not.

## Clinical implications

From a clinical and translational perspective, these findings suggest that raising HDL-C per se should not be a primary therapeutic goal unless accompanied by demonstrable improvements in HDL functionality and overall cardiovascular risk. Exercise remains a uniquely effective intervention, as it improves HDL function in a biologically integrated manner, even when HDL-C changes are modest. Conversely, the experience with CETP inhibitors underscores the limitations of targeting HDL-C levels without fully capturing its functional and systemic roles. In summary, exercise produces smaller but more physiologically coherent and clinically meaningful effects, whereas CETP inhibitors highlight a dissociation between HDL-C elevation, HDL functionality, and cardiovascular outcomes. While the final verdict on CETP inhibitors remains pending, exercise should be prescribed as a cornerstone of CVD prevention, owing to its favorable effects on blood pressure and glucose metabolism, thereby reducing cardiovascular risk.
